# Forming ideas about health: A qualitative study of Ontario adolescents

**DOI:** 10.3402/qhw.v10.27506

**Published:** 2015-05-25

**Authors:** Valerie Michaelson, Margaret McKerron, Colleen Davison

**Affiliations:** 1 School of Religion, Queen's University, Kingston, Ontario, Canada; 2 Department of Theological Studies, Regent College, Vancouver, British Columbia, Canada; 3 Department of Public Health Sciences, Queen's University, Kingston, Ontario, Canada

**Keywords:** Adolescent health, health perceptions, paediatric, organic learning, didactic learning

## Abstract

Adolescence is a crucial period of child development during which one's ideas about health are formed. However, little is known about the different contexts, experiences, and potential other factors that contribute to shaping the health ideas of adolescent populations, particularly when they are not seeking out the information for a particular purpose. In this Ontario-based qualitative study, grounded theory methods were used to explore ways that health knowledge is obtained in adolescents (age 10–16). A purposeful, criterion-based sampling strategy was used, and data were collected through seven focus groups (*n*=40). Findings indicate that while young people get their ideas about health through both didactic and organic learning contexts, the significant impact of organic learning is often overlooked. Categories of organic learning that emerged include self-reflective experience, the experience of close contacts, casually observing others, and common discourse. This study suggests that one central way that young people get their ideas about health is from living life: from the people they watch, the conversations that they have, and the experiences they live. Findings support the development of effective health promotion messages and also contribute to considering the place of some aspects of organic learning in the development of health-related resources that target adolescent populations.

Adolescence is a crucial period of child development during which one's ideas about health are formed. These lifelong beliefs and practices relate to significant health risks and protections throughout adulthood (Holmbeck, Williams, & Greenley, 2002; Woodgate & Leach, 2010). To provide effective support for health and well-being for this age group, it is useful to examine adolescents’ perceptions of how their own ideas regarding health have been obtained and formulated. Using an Ontario-based sample of adolescents, this study therefore explores the question “Where do young people get their ideas about health?” By taking seriously the perspectives of adolescents, it aims to contribute both empirically and theoretically to appreciating the multifaceted ways that shape adolescents’ ideas about health. This includes exploring ways that their attitudes, perceptions, and views about what constitutes healthy behaviour are obtained and formed.

Specific conceptualizations of health are not the issue of interest for this particular investigation. Health is understood in terms of what participants themselves perceive to be healthy behaviours and attitudes, based on their own information, experience, and opinions. Previous research suggests that when information about a particular health issue is wanted, adolescent populations have preferred resources for attaining relevant information (Ackard & Neumark-Sztainer, 2001; Marcell & Halpern-Felsher, 2007). Depending on the issue, these include parents and in particular mothers (Ackard & Neumark-Sztainer, 2001), peers (Marcell & Halpern-Felsher, 2007), the Internet (Beamish et al., 2011; Selkie, Benson, & Moreno, 2011), and less centrally, doctors and healthcare practitioners (Ackard & Neumark-Sztainer, 2001; Leavey, Rothi, & Paul, 2011). Furthermore, when adolescents choose to access health-related information, they have identified trusting relationships, reliable resources, and privacy as significant concerns (Smart, Parker, Lampert, & Sulo, 2012). However, little is known about the different contexts, experiences, and potential factors that contribute to shaping the health ideas of adolescent populations. By focussing on where adolescents perceive obtaining ideas about health, we hope to contribute relevant guidance for effective approaches to adolescent health promotion. In addition, we hope to illuminate ways that traditional models of health-related teaching may be strengthened or complemented to become more efficient for today's young people.

## Methods

This study design was inspired by the qualitative, constant comparative method of grounded theory, developed by sociologists Glaser and Strauss (1967). In this approach, researchers begin the project with an area of interest, rather than a preconceived theoretical perspective, and by adhering to this principle, the particular research problem and the theory to account for the social phenomena are conceived “from the ground up” (Charmaz, 2014, p. 125). We began with a general interest in youth perceptions of health, and data were generated as we asked open-ended questions related to this topic. Central to this method is simultaneous involvement in both data collection and analysis. Because the researcher engages in analyses in the midst of the data collection process, mid-collection analysis shapes subsequent data collection procedures. As theories begin to emerge throughout initial coding and analyses, subsequent questions are then modified and become more focussed. Earlier data allow for the researcher to compile more evidence around emerging themes and questions. In the early data collection efforts of this broader study of health and its perceived determinants in young Ontarians, a significant theme emerged regarding where young people get their ideas about health. The interviewers focussed questions iteratively in order to explore this theme more fully throughout subsequent data collection. By staying close to the data through constant comparison and memoing, the theory resulting from this iterative process was grounded in the data.

### Sampling selection and recruitment

A purposeful, criterion-based sampling approach was used. To facilitate conversation, this initial sample aimed to recruit homogeneous groups, with diversity achieved between groups rather than between individual participants within a group. This was accomplished by recruiting participants who shared one or more criteria to participate in the same group. These criteria included age, sex, rural/urban, and newcomers to Canada/ethnic. These groups were also diverse geographically and were selected from populations in Eastern Ontario (Hastings and Frontenac Counties), Northern Ontario (Greater Sudbury), Western Ontario (Bruce County), and the Greater Toronto Area. Our final sample included seven groups.

A total of 40 young people were involved. All participants were recruited using “snowball” or “chain” sampling (Patton, 2001). Letters of information and informed consent were given to well-situated people whom we predicted might be aware of potential participants, with the request to circulate the study information in their community. Consistent with the grounded theory methodology, sampling did not aim for population representativeness but aimed towards theory construction. Recruitment was considered complete when theoretical sampling had been achieved and the core question was saturated. Saturation does not imply that novel ideas would not emerge with additional data collection; rather, it suggests that sufficiently rich and dense data have been collected to enable an adequate understanding of key concepts (Charmaz, 2014).

### Data collection

Data collection was performed via semistructured interviews of seven focus groups. Focus group methods were an especially appropriate methodology for this study in that they are highly flexible and practical, permitting the gathering of large amounts of information in relatively short periods of time, and may reveal concepts that previously have not been considered by the researcher (Babbie, 2007). Furthermore, focus groups enable researchers to better understand how members of a group arrive at, or alter their opinions or conclusions about, some topic or issue as they communicate. The facilitator can explore related but unanticipated topics as they emerge, and they do not require complex sampling strategies (Patton, 2001). Furthermore, potentially sensitive topics may appear to be less threatening to participants when activities and tasks are incorporated into the focus group sessions (Berg, 2009). One sample question asked was this: “When you think of a healthy person, what is one of the first words that comes to your mind?” To invite rich discussion, the focus groups involved core, open-ended questions that were asked of each group as well as the opportunity to interact with standard definitions of health. For example, they were asked, “Are there any words you would like to add or take away?” and “Do any of these definitions resonate with you more than others?”

Photo elicitation techniques were also integral to this purposeful strategy (Harper, 2002). Photo elicitation is a research technique that “enlarges the possibilities of conventional empirical research” (Harper, 2002, p. 13). The key element in photo elicitation is “not the form of the visual representation, but its relationship with the culture under study” (Harper, 2002, p. 19). A series of small cardboard cards containing photographs were developed. Themes and images depicted in these cards were meant to elicit responses surrounding well-known categories of health, including its physical, mental/emotional, social, and spiritual aspects. Photographs that might elicit conversation about the contexts that young people are exposed to and could influence and determine health were also chosen. These included images depicting aspects of poverty, affluence, school environments, home environments, and neighbourhoods, and images showing the larger environmental context of our planet. Sixty images were selected by the researchers and presented to a pilot group of adolescents prior to the study. The 40 images that generated the most meaningful conversation during the pilot group were then selected for the study. The interviewers provided an opportunity for participants to reject any of the cards if they did not feel that they related to health. They were also given an opportunity for new and unanticipated themes to emerge from the discourse. A sample question used for this activity was “Do any of the cards that you have help you to describe any aspects of being healthy or unhealthy?”

Although photographs do not automatically elicit useful conversation, when used effectively, they have the power to capture an element of human consciousness or experience that is different from and complementary to “words-alone” interviews. This has been attributed to the way that remembering is enhanced by visual prompts (Harper, 2002). Because using predetermined photos introduced an element of preformed ideas about health into our study, this study is described as “grounded theory inspired” in that it lacks the complete openness of grounded theory. Regardless, even when using predetermined images, participants will see the images subjectively and bring their own experiences and ideas into the conversation (Flick, Von Kardorff, & Steinke, 2004). Photo elicitation is a powerful tool in that it may further facilitate meaningful conversation by anchoring the conversation in an image that is understood, at least in part, by all parties and is external to any one individual. This allows new interpretation and different perspectives to emerge. In the initial phases of the study, the broad question “Where do you get your ideas about health?” was asked and discussed among young people. Participants gave fairly predictable answers, such as “from parents,” “teachers,” and “health class.” However, we observed that as the discussion progressed, participants often identified experiences that had shaped their ideas about health that were implicit to their own life experiences. For example, participants often shared about their experiences with parents and friends. Repeatedly, a participant would consider a particular experience and reflect on how their own thinking had changed or been shaped through it. We became interested in this theme early on, and iteratively, our methodology enabled us to probe this more deeply by asking questions such as “Can you help us to understand where you got that opinion or idea about health?” or “Can you tell us about how that experience shaped your current ideas about health?”

### Coding and analysis

Focus group interviews were transcribed verbatim and examined consecutively line by line in order to identify each participant's descriptions of thought patterns, feelings, and actions that emerged throughout the interviews. Codes were formulated from emergent themes and were then compared to verify their descriptive content and to confirm that they were grounded in the data. Data were coded (or organized into key conceptual themes) primarily by two researchers. At first working independently, these two researchers reviewed several transcripts in order to determine preliminary coding structures for organizing the data thematically. Initially, open and descriptive codes were applied. A second level of axial coding was then applied, which resulted in the identification of higher level, more conceptual categories. At this point, the two main themes of *didactic learning* and *organic learning* began to emerge. We focussed on these two themes and applied a third level of selective coding to identify any patterns within the underlying ideas or concepts. During this third level of coding, we focussed on identifying specific themes related to didactic and organic learning that emerged throughout the interviews. This multistage approach to coding helped to provide an in-depth understanding of how young people learn about health.

### Academic rigour

To ensure that the codes and categories being developed remained grounded in the data, the grounded theory tools of constant comparison, theoretical sensitivity, and triangulation of researchers were employed during the entire process. This included being sensitive to the literature base in this area and being purposeful about separating what was pertinent in the data from what was not. Furthermore, as ideas emerged, they were compared with previous and subsequent interviews. The interview guide was modified iteratively based on the analysis of previous interviews, which was done in order to probe more deeply into the codes and theories that were emergent. Theoretical memoing was used throughout the coding and analysis process in order to record additional insights, questions, and themes. Multiple researchers (one faculty member, two postdoctoral fellows, and two students) were involved in data collection and analysis, and coding was discussed and compared across researchers to provide the multiple perspectives that help improve rigour. At the third level of coding, an additional researcher (CM) was consulted in order to further enhance the rigour of the coding and associated analytic process. Confirmability was also ensured by maintaining an audit trail of all analytical memos and revisions to the coding structure. Two coders (VM and MM) applied the final coding structure to the complete data set, using qualitative data analysis software (Dedoose Version 5.0.11, Los Angeles, CA) to facilitate cross-referencing.

### Ethical considerations

This study received ethics approval from the Health Sciences Ethics Board of Queen's University (approval number EPID-447-13Romeo #6011166). Participants and parents were given verbal and written information about the study and a telephone number to contact if parents had more questions about the study. All parents gave written, informed consent prior to the study, and all participants provided written informed assent prior to the study and verbal informed assent at the time of the study.

## Results

The study population is described briefly in Table I. All participants (*n =* 40) were fluent in English and all attended public schools in Ontario. All participants responded to the request for participation with mediation and approval by their parents.

**Table I:** Demographic characteristics of the study population.

Gender	
Boys	13
Girls	27
Age (years)	
12–13	22
14–15	18
Size of community	
Large urban centre	13
Medium-sized town	18
Rural locations	9
Total participants	40

**Figure 1:**
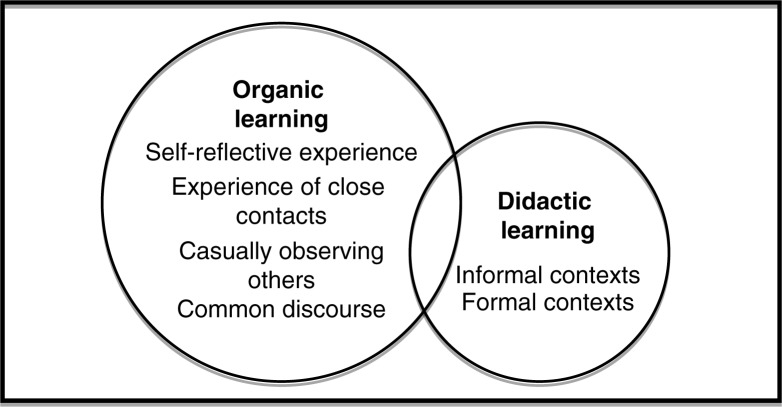
Core categories and subcategories of participant learning about health.

Analysis of the focus group findings provided a detailed description of ways that the sampled adolescents learned about health. With respect to the recurrent themes that arose in our analysis, the implicit and explicit ways that adolescents internalize health knowledge consistently emerged as important in all focus groups. For the purpose of this study, we call these two themes: *organic learning* (implicit) and *didactic learning* (explicit) (Figure 1).

By *organic learning*, we mean implicit learning that occurs through experiences that arise naturally throughout the course of everyday life. A large body of research reflects a growing interest in organic learning in areas as diverse as education (Nedovic & Morrissey, 2013), architecture (Moller, Prestera, Harvey, Downs-Keller, & McCausland, 2002), management (Beckett, 1999, 2000; Pittaway & Cope, 2007), nursing (Swallow & Macfadyen, 2004), and human resources (Beattie, 2007). Organic learning can happen in any situation (Mahar & Harford, 2005), and the term is used to describe learning that takes place in a natural setting, without being contrived (Beckett & Hager, 2000; Moss, 2000). Throughout the focus groups, we observed that such organic learning occurred in four ways: through self-reflective experiences, through experience with a close friend or family member, by casual observation of others, and through interaction with ideas in the common discourse. By *didactic learning*, we mean more explicit learning that occurs in both formal contexts (e.g., school and health class) and informal contexts (e.g., “My Mom always tells me to …”) in which deliberate teaching is intended. For example, in more formal contexts, such as health class, the teacher intentionally conveyed information to the student(s) (often involving a curriculum or lesson plan). In more informal contexts such as the home, it did not seem likely that a parent was following a formal “lesson plan”; however, based on reports from these participants, parents appeared to be intentional about conveying particular messages about health to their child drawn from their own repertoire of health knowledge. As shown by the size of the two circles in Figure 1, organic learning was repeatedly identified by participants as much more influential in shaping their ideas about health than the learning that took place in didactic contexts. Several of the examples shared by participants demonstrated that organic learning and didactic learning can occur in the same situation (i.e., a gym teacher giving a lesson about health while simultaneously modelling a healthy lifestyle).

### Examples of organic learning

#### Self-reflective experience

Organic learning appeared to occur informally when a person reflected on his or her own experiences and/or internally examined issues of concern or interest, which in turn created potential new meaning. For example, participants repeatedly reflected on how their personal use of technology had shaped their ideas about the connection between technology and health. One participant observed,

I used to have [my phone] all of the time and I would be really angry and stuff. And then my mom was like … [pause] and I thought I was happier and it was easier …. There was a lot of drama that was involved with it. It made me more stressed and when I didn't have it I felt better.

Another reflected, “When I spend a lot of time on electronics I get a bad headache.” This theme of considering one's own experience also emerged as participants talked about other aspects of health. Sleep was reflected by a subsequent participant:

It is hard to function without sleep and also when your body grows it develops mostly when you are sleeping and resting. Even at school and during exam times I want to stay up late to study. I was writing an English essay and stayed up really late writing it and the next morning I was reading it and I was like, what is this? When you are tired you just don't function as well.

This self-reflective type of experience did not solely occur in relation to negative health outcomes. The next participant appeared to imagine an experience he might have around helping others. He said,

If you are helping other people then you could say something that you had not thought of before that could help them but also help you. So something could pop into your head and I am helping someone but it also helps myself.

#### Experience of close contacts

Organic learning seemed to occur informally when participants were exposed to particular experiences or circumstances involving a close contact, such as a family relation, friend, or teacher. Family formed the most important context for informal, organic learning for many participants because, as one participant remarked, “Your family is where you spend most of your time and they are the first people to influence your thinking.” Specifically, participants acknowledged that “your parents set an example of what a healthy lifestyle is.” When participants postulated hypotheses for unhealthy behaviours such as smoking or bullying, they routinely referred to parental modelling as an explanation (i.e., “If your family smokes and everyone you know smokes, then you will probably start to smoke because it is what you grew up with”).

This modelling was also significant in shaping health-related ideas in more formal contexts, such as at school. One participant shared this story about his health teacher: “I had a teacher last year who really lingered on health discussions. He was a fit guy and so he taught us bare bones what it means to be healthy.” When asked if the observation that he was a really fit guy made a difference to the participant when he was listening to what the teacher had to say, the participant said,

Well yeah. He also taught us that if your health teacher tells you not to smoke but every day you see him go off to the parking lot to smoke a pack it doesn't really … it doesn't have the same meaning.

Relationships with peers represent another significant context for informal, organic learning about health through observing the experiences of close contacts. Specifically, participants frequently mentioned watching friends go through mental health challenges such as depression and anxiety or struggles with body image. They repeatedly reported that these were situations that shaped their ideas about health.

#### Casually observing others

Organic learning appeared to happen through casually observing others with whom participants did not appear to have any particular, direct relationship. For instance, one participant remarked that she gets her ideas about health “mostly from what I know of my friends or people I see at school or on the street just walking around. Just by watching people and seeing how they act.” School was a notable site of general casual observation (e.g., “When observing people at school, your brain automatically puts people into categories of healthy and non-healthy people, just by observing”). Specific knowledge about diseases, such as diabetes and cancer, was usually associated with “knowing someone” who had them. An interesting dialogue took place between two participants who were reflecting on why they had said that smoking was unhealthy. The first said,

It is bad for your lungs because you are breathing in the bad stuff, and it is unhealthy for the environment because usually people just throw it on the ground. And it is unhealthy for the people around you because of the smoke coming out.

A co-participant then continued to speculate, based on her own observations,

I think of where did he get it and how was he motivated to actually do it? I feel that for someone that age, I know people who do that … I see them and always wonder what is their story? Did they have parents that smoked or are they peer pressured into it or something? I also think … especially if they look nervous or something and then it is like maybe there is some sort of a clique that they wanted to join and they are like, you have to show that you are worth it and you have to go and smoke even if your parents don't want you to.

These participants used their casual observations of peers not only to shape their own ideas around the negative health impact of smoking but also to reflect on the environmental factors that might contribute to making a decision to smoke in the first place. Although casual observation of others did appear to influence participants’ ideas about health, it is important to note that this category was the weakest of the four categories related to organic learning.

#### Common discourse

Organic learning about health appeared to happen through engagement with ideas circulating in common discourse (i.e., the body of beliefs, values, and ideas related to health shared by a community). Participants almost always used the unspecified “people” or “everyone” to introduce such tacit ideas about health. Generally, these ideas related to physical well-being, especially being active. For example, participants said, “People associate being active with being healthy, and so people who are constantly active … there is an image of that being healthy” or “At school, everyone is saying that it is important to be active and to be healthy.” Referral to this common discourse happened repeatedly throughout all groups and appeared to reflect a canon of common knowledge that participants generally agreed on, even though they could not point to a particular source, for example: “When I was younger people would say, you should play outside and do a sport rather than be inside.” Another said, “I don't know how I get the information [about health], but I just hear about it I guess.”

### Examples of didactic learning

#### Formal contexts

We conceptualized the second emergent theme as *didactic learning*. This related to learning that occurred when deliberate, intentional instructions or information were given to the participants. We identified formal contexts as those that might be expected to be used formally to convey health-based information, such as school health classes or doctors’ offices. When participants were asked, “Where do you get your ideas about health?” such contexts were often identified. Ideas about physical well-being, especially about healthy eating, sexual health, and substance abuse, were reportedly shaped in this kind of formal context, usually in a school-based health class. Aspects of social well-being, such as healthy relationships and bullying, were also recognized as being learned about in formal, didactic contexts, although this was less common than learning about aspects of physical well-being in these contexts.

When asked about the impact of health class on influencing ideas about health, responses were mixed. One participant used the exact words of the Ontario health curriculum to define health, and another participant affirmed its positive impact. However, a third participant related, “I listen to music in health class. I think it is good for a certain amount of years but once we have covered everything it is not as necessary.” This third experience was quite typical. Another participant said that what she learned in health class was “more for school” than in other life contexts. Participants commonly noted the typical approach of reviewing material and standard curriculum that characterize their health classes (e.g., “just a bunch of review”) and its irregularity (e.g., “We barely did health this year”). Interactions with health practitioners, such as doctors and nurses (in both school and other contexts), were also cited in nearly half of the focus groups. Irregular contact was also acknowledged in this context (“I don't go to the doctor that frequently, so they would not have an impact on the way that I think”).

#### Informal contexts

Intentional and deliberate didactic teaching around health ideas also occurred in more informal, everyday living situations, particularly in the home. Parents were reported to provide considerable influence on health knowledge, such as healthy eating choices (e.g., “Eat all your food groups”) and habits of moderation (e.g., “My mom will say, eat it and be conscious of what you are eating. Otherwise, you eat so much and you won't know how much you take in”). These were prevalent themes for instruction; teaching around technology use was also common. If a specific parent was identified as teaching about some aspect of health, it was almost always the mother, and some version of the refrain “My mother always says …” was present in multiple focus groups. For example, one participant said,

My mom always says that if I am sick … recently I just had a bad cold and she said if you are sick then you need to sleep so I went to bed much earlier. And sometimes I missed part of school in the morning so that I could sleep and get better. Sleep is the best way to get better.

The only time “my dad” was cited as a source of health knowledge was once in the all-boys focus group. Although less common, participants also intentionally sought out health-related advice or instruction from older figures outside the family; coaches were cited on several occasions, sometimes with some reservations (“I guess you could talk to them about stuff, but you probably would not get as good advice back because they don't know you as well as your friends and family who know you”). Others included camp counsellors and older church members whom “you might not see as often but you can trust … and talk to about anything.”

These didactic teaching opportunities, whether they occurred in a formal context (such as school or a doctor's office) or in an informal context (such as mother's teaching about eating practices), were identified as somewhat important in providing a basis for young people to learn. Nevertheless, within formal contexts related to health education, these didactic teaching opportunities were not reported to have a significant impact on shaping young people's ideas about health. Within the context of the home, however, parents’ comments about health practices appeared to be very significant (mothers’ in particular).

Although didactic and organic learning were both sources of learning about health for adolescents, it appeared that the learning that took place in organic, real-life contexts was the most influential in shaping ideas about health. In other words, relationships, observation, and direct experience appeared to be the most powerful ways that young people absorbed health knowledge. This was evidenced by the number of times that participants told stories about health knowledge that they had learned in organic contexts (“I have a friend who …”) and also by the emphasis they themselves put on these experiences. On many occasions, there was overlap between organic and didactic learning (e.g., the mom who exercised with her child and talked about healthy activity, and the health teacher who was physically fit). In these contexts, it appeared that the didactic learning was strengthened greatly by the modelling.

## Discussion

In this qualitative study, we had the opportunity to explore adolescent perceptions of health and the ways that health knowledge is obtained. The qualitative findings indicate that there are two main ways that young people gain ideas about health: through didactic learning and through organic learning. Although we are not suggesting that didactic contexts, both formal and informal, are unimportant venues for learning about health, the organic ways that adolescents get their ideas about health consistently emerged as an important issue and powerful source of knowledge in all focus groups. The importance of this theme of organic learning was signalled not only by its recurrence but also, more importantly, by the ways these ideas came up implicitly throughout the interviews, even when this topic was not what was being asked about. Thus, the importance the young people gave to organic modes of learning about and internalizing health knowledge became a salient theme in this study. The categories of organic learning included self-reflective experience, the experience of close contacts, casually observing others, and common discourse.

### Self-reflective experience

Reflective learning is understood to be “the process of internally examining and exploring an issue of concern, triggered by an experience, which creates and clarifies meaning in terms of self, and which results in a changed conceptual perspective” (Boyd & Fales, 1983, p. 99). This kind of learning was evidenced many times during our focus groups. For example, one participant reported,

My mom always says if I have my phone, this is time for family and not for you and your other 15 friends that you are talking to right now. That is true …. If you are always on your phone then you can't be like, how was your day and what are you or plans? You are in your own world and with your friends you are probably going see another time and you are not valuing the time spent with your family.

This is an example of how what her “mom always says” enables her to reflect on her own experience. This echoes other research that has demonstrated that learning from experience happens during a reflective process that has the potential to challenge and change perspectives (Boyd & Fales, 1983; Domke-Damonte & Keels, 2015). For example, this kind of strategy has been used effectively in business contexts involving the intentional combining of reflective practices with simulation games. Students are guided to solve team problems by using reflective practices such as reflecting on past experiences, and the results of this process have been shown to be beneficial to overall learning (Wills & Clerkin, 2009). By incorporating self-reflective questions and practices into the curricula and other health-related resources (including nonconventional games and apps), adolescents could be encouraged to adopt intentional reflective practices that maximize the potential of their own personal experience as a means of shaping ideas about health.

### Experience of close contacts

The second major category that was identified as shaping adolescent ideas about health was related to observing the experiences of close contacts such as a parent, teacher, or close friend. As reported in the “Results” section, one participant clearly identified that, when his very fit health teacher taught him “bare bones” what it was to be healthy, he knew he could trust what was being said because the teacher's actions emulated his words.

Although the importance of positive role modelling was consistently emphasized, health-related experiences with close contacts did not need to be positive to make a positive impact on the health ideas of participants. For example, this participant provided an animated description of what he perceived to be his mother's negative physical health. He said,

My mom and I were doing [a marathon last June] and we have not been physically fit all this winter. Well I have but my mom hasn't. We went walking 8 km and she got back to the house drenched in sweat and huffing and puffing and I was still in the driveway ready to go again. I looked at her and said, what is wrong with you? You go to the gym and you are not fit. I just walk every day and all that stuff.

This participant was learning from observing his mother, even though he considered her health behaviour around physical activity to be less than optimal. Participants told us, “Your family are the first people to influence your thinking.” Watching people close to them, particularly teachers and close family members, seemed to shape ideas about health in participants, and important to this category is that the people who were being observed as role models, and facilitated organic learning through observation, were also people who were involved in didactic teaching (e.g., health teachers, or moms giving intentional advice about nutrition, sleep, or physical activity to their child). What was poignant in this category was that the didactic learning was received more strongly when the organic learning was observed. Here, two interesting yet contradictory themes emerged: the health teacher was taken more seriously because he or she “walked the talk,” and yet the mom who was out of shape still gave an important health message, presumably because her child had first-hand experience of the consequences of lack of physical activity. For participants, observing close contacts influenced ideas about health regardless of whether or not the health behaviour being observed was positive or negative.

Parents are usually unconscious of their individual roles and of their potential consequences on health and development (WHO, 2007). Moreover, Sanderse (2013) argued that teachers rarely use role modelling as an explicit teaching technique and that only a very small percentage of adolescents recognize teachers as role models (Sanderse, 2013). For teachers, Lunenberg, Korthagen, and Swine (2007) suggested that even though they may want to be good role models, they often lack the skills needed to make their own teaching and behaviour in the classroom connect more explicitly with their ideals. This may be true for parents as well. For teachers, parents, and others in the position to be role models, by better understanding their own positions as role models and influences in the lives of young people, they could more intentionally and attentively model healthful attitudes and practices to young people. Lumpkin (2008) argued that teachers can and should serve as role models in a school setting and should use their influential role to benefit the overall well-being of young people. Perhaps even more so than teachers, “Families are the most central and enduring influence in children's lives” (Schor, 2003). Parents clearly have a critical role to play in the health knowledge and behaviours of their children, particularly because they are the primary forces shaping the environment in which their children engage with the world and the environment in which organic learning can occur. Parents’ own modelling of these practices provides vital elements of a template for providing life-long health information and mapping behaviours to adolescents. Resources could be developed to target parents and teachers, encouraging them to take seriously their important role as role models who help to shape ideas about health in adolescent populations.

### Casually observing others

The third category that emerged related to how adolescents get their ideas about health was that of observing others who were not close contacts. This category shared some similarities with category 2, which has been discussed in terms of role modelling of close contacts, in that both were concerned with what was learned in terms of observing others. However, this category had its own distinct qualities in that the participants did not have much information about the stories of the people who were being observed. Although many participants identified casually observing people as significant, what they shared in the context of these more casual observations had much less detail than the stories they shared about people they know well. Upon reflection, it is not surprising that young people report learning from watching those around them. Indeed, Bandura (1997) described the way that watching the actions of another person reinforces one's own actions. Bandura draws on a large and early body of literature described as *social learning theory* (or social cognitive theory), which suggests that it is by observing what others do that people learn (Miller & Dollard, 1941). This thinking has been developed in more recent educational psychology, and the unpredictable nature of reinforcement and punishment on both behaviour and learning has been explored (Ormrod, 2003). This literature is of relevance to this study because it suggests that there is not a universal outcome that can be expected from observation. For example, if one person observes a group smoking outside their school, their perception might be that it is an unhealthy behaviour. However, for another, it might signal a good communal experience and be considered a positive health behaviour. This category was the least stable of all the ways that participants reported that they learn about health. Although this category emerged as important enough to note, the depth of what they learned through casual observation was not nearly as significant to shaping their ideas as what they learned from reflecting on their own experiences and by observing closer contacts who could serve as role models.

### Common discourse

Fourth, and finally, young people identified “common discourse” as a way their ideas about health are shaped. There appeared to be an accepted “canon” of health knowledge that was generally tacit, but when it came into the conversation signalled by phrases such as “Everyone knows” or “People say …,” it was unanimously accepted by the group at hand. This also relates to literature about *social norms* that demonstrates the power of a commonly held set of beliefs within adolescent population groups around various health behaviours (Carter, Bingham, Zakraisek, Shope, & Saver, 2014; Eisenberg, Newmark-Sztainer, Story, & Perry, 2005). Discourses on health are not arbitrary or random. Rather, these discourses emerge and gain acceptance because they are in keeping with the prevailing attitudes of the context and culture in which they are produced. Because discourses about health are always attached to other interests and agendas, Robertson (1998) argued that when we conceptualize, speak, and write about health, it is never only about health. Indeed, these discourses also echo our ideas about human nature and society, and furthermore, an examination of “particular discourses on health, therefore, provides a unique window onto these deeper ideas and beliefs” (Robertson, 1998, p. 155).

An example of how understanding discourses has been effectively used as a health intervention is demonstrated in a study by Ristovski-Slijepcevic, Chapman, and Beagan (2008) regarding healthy eating discourses. In this study, engagement with different discourses led participants to undertake different personal practices in the name of healthy eating. Intentional engagement with a variety of discourses around health could, in a similar way, potentially help young people understand and evaluate their own ideas about health, and in turn adopt thoughtful health ideas that would in turn lead to positive health behaviours. Future research that leads to understanding a specifically adolescent discourse on health could be valuable. This could be used not only to promote positive health ideas but also to identify ideas about health in this population group that potentially lead to unhealthy behaviours and decisions. These could in turn be targeted as interventions.

### Didactic learning (formal and informal contexts)

Along with the four areas of organic learning that have been discussed, didactic learning (in both formal and informal contexts) was also identified by some participants as important in shaping adolescents’ ideas about health and must not be undervalued. Indeed, there is an important role for intentional, didactic resources such as health curriculum, web, and other resources that target adolescent populations. The discussion in this paper has disproportionately focussed on the aspects related to organic learning because those areas were clearly identified by all participants as the most important ways that they get their ideas about health. We argue that it would benefit adolescent populations if attention were given to the organic ways that health information and ideas are internalized and obtained.

## Next steps for interventions and research

This study suggests that one central way that young people get their ideas about health is from living life; from the people they watch, the conversations they have, and the experiences they live. Findings support the development of health promotion messages that target strategically placed adults (parents, teachers, coaches, etc.) in order to help them understand the extent to which they are being watched and emulated by the adolescents around them. Furthermore, when didactic resources are developed, it would be worth considering the place of encouraging self-reflection on one's own health practices in these resources.

In terms of research, although a link between perceptions and behaviour in adolescent health choices can be inferred, this has not been clearly established in the literature. This could be studied with the goal of understanding the causal relationship between perceptions, behaviours, and health choices in adolescents. This would demonstrate whether or not perceptions or ideas about health actually determine health behaviours. Furthermore, although discourse analysis is a way of understanding population groups, and more specifically the area of health promotion (e.g., the changing discourse in public health promotion; Porter, 2007), the health discourse in adolescents has not been comprehensively studied or described in a present-day context. This would include what it is, how it is shaped, and how it does or does not lead to healthy behaviours. Such research would provide essential information to direct the development of future health resources and health promotion messages directed at adolescents. This could be done by either affirming or challenging the common discourse, or social norms, that are being adopted by this population group.

## Summary

Although there are many valuable ways of learning about health, our findings suggest that experiences that occur within the context of everyday living are powerful in terms of shaping health ideas in adolescent populations. Although explicit, didactic learning about health is not unimportant, it is by reflecting on their own experiences, by watching others (both close contacts and casual observation of people), and by “living life” that young people are learning the lessons that remain with them most strongly.

### Strengths and limitations

Strengths and limitations of our study warrant comment. With respect to limitations, this study was limited geographically in that all participants were representatives of one Canadian province. The use of focus groups as a research tool also has many limitations. First, focus group results are not generalizable to a full spectrum of young people in Canada or other countries. Also, participants could engage in only one focus group on a single day. Thus, our results do not take into account how their experiences might be articulated at a different time, what their experiences might be during a different season of life, or what their experiences might be after they have matured through natural development. Another limitation of focus groups in general is that they may be dominated by one or two opinions, and others may not share their own opinions honestly. This may have occurred and impacted the accuracy of our findings. This danger was, however, mediated by the experienced facilitators who guided the groups because they were more likely to be able to adapt to this kind of challenge. A third danger of focus groups is around sample bias in that focus group participants are often recruited from a limited number of sources (Morgan, 1996). However, because this study is based on recruitments from seven different sites across Ontario, that danger has been minimized. Finally, because all participants responded to the request for participation with mediation and approval by their parents, this may have led to a positive bias in terms of positive parent—child communication or relationship in that participants without a positive relationship with their parent(s) may have declined participation.

Despite such limitations, the results of our research make a useful contribution to understanding how adolescents get their ideas about health, and ensuring that Ontario youth have a voice in adolescent health research is essential to gleaning a full picture of this type of health research. Notable strengths of our study include the richness of the qualitative data that emerged. Participants in the focus groups were articulate and engaged. The resultant data are textured, deep, and nuanced, and observations were honestly and generously shared. Participants came from a variety of age groups, and they represented the views of both genders and a diversity of school and family experiences. Participants were also eager to share, and the facilitators were experienced and trained in qualitative methods. Finally, our study is based on questions with significant practical value as they are rooted in honest, real-life experiences.

## Conclusions

Findings from this study raise important questions about where young people get their ideas about health. Although it is certain that intentional, didactic teaching is an important component of communicating health knowledge, the impact of organic learning is often overlooked. Organic learning, in the context of everyday life, is clearly a central way that adolescents receive health knowledge. Home and school contexts are primary locations for organic health knowledge to be absorbed. Intentional reflection on the impact of organic learning by adolescents, and the reality that what they do is much more important than what they say, will enable teachers, parents, and others to be more proactive in creating healthful environments for today's young people.
